# *IRS-1* gene polymorphism and DNA damage in pregnant women with diabetes or mild gestational hyperglycemia

**DOI:** 10.1186/s13098-015-0026-3

**Published:** 2015-04-02

**Authors:** Rafael B Gelaleti, Débora C Damasceno, Daisy M F Salvadori, João Paulo C Marcondes, Paula H O Lima, Glilciane Morceli, Iracema M P Calderon, Marilza V C Rudge

**Affiliations:** Department of Gynecology and Obstetrics, Botucatu Medical School, Unesp_Univ Estadual Paulista, Laboratory of Experimental Research in Gynecology and Obstetrics, Distrito de Rubião Júnior s/n, CEP. 18618.000, Botucatu, São Paulo Brazil; Department of Pathology, Laboratory of Toxigenomics and Nutrigenomics, Botucatu Medical School, Unesp_Univ Estadual Paulista, Botucatu, Brazil

**Keywords:** Diabetes, Mild gestational hyperglycemia, Pregnancy, Newborn, Polymorphism, DNA damage

## Abstract

**Background:**

Pregnant women with mild gestational hyperglycemia present a high risk for hypertension and obesity, and appear to reproduce the model of metabolic syndrome in pregnancy, including hyperinsulinemia and insulin resistance. Diabetic patients have a higher frequency of the IRS-1 Gly972Arg variant and this polymorphism is directly related to insulin resistance and subsequent hyperglycemia. In diabetes, hyperglycemia and other associated factors generate reactive oxygen species that increase DNA damage. The aims of this study were to evaluate the presence of the IRS-1 Arg972 polymorphism in pregnant women with diabetes or mild gestational hyperglycemia, and in their newborns. Additionally, we evaluated the level of primary DNA damage in lymphocytes of Brazilian pregnant women and the relationship between the amount of genetic damage and presence of the polymorphism.

**Methods:**

A based on the oral glucose tolerance test (OGTT) results and on glycemic profiles (GP), as follows: non-diabetic group, mild gestational hyperglycemia (MGH) and diabetic group. Eighty-five newborns were included in the study. Maternal peripheral blood samples and umbilical cord blood samples (5–10 mL) were collected for genotyping by PCR-RFLP and for comet assays.

**Results:**

The prevalence of genotype Gly/Arg in pregnant women groups was not statistically significant. In newborns, the frequency of Gly/Arg was significantly higher in the MGH and diabetic groups than in the non-diabetic group. Taken together, groups IIA and IIB (IIA + IIB; diabetes) presented lower amounts of DNA damage than the non-diabetic group (p = 0.064). No significant association was detected between genetic damage and the presence of the Arg972 genotype in pregnant women.

**Conclusion:**

The polymorphism was more prevalent in newborns of diabetic and MGH women. We believe that it is necessary to increase the number of subjects to be examined in order to better determine the biological role of the Arg972 polymorphism in these patients. Despite being classified as low-risk, pregnant women with mild gestational hyperglycemia characterize a population of maternal and perinatal adverse outcomes, and that, together with their newborns, require better monitoring by professionals and health services.

## Introduction

Maternal hyperglycemia in pregnancy is associated with adverse outcomes, including birth weight ≥90th percentile, delivery by cesarean section, neonatal hypoglycemia, and fetal hyperinsulinemia [1]. These associations occur across the full range of increased maternal glucose levels, including those below that classified as overt diabetes [1]. In fact, our previous findings have shown that not only gestational diabetes mellitus (GDM), but also mild gestational hyperglycemia (MGH) is responsible for adverse perinatal outcomes (APO), such as high incidence of macrosomia, perinatal mortality, hypoglycemia, hyperbilirubinemia, prematurity, and congenital anomalies [2]. MGH is a risk factor for metabolic syndrome (MS) in pregnancy. Patients with MGH have hyperglycemia, hyperinsulinemia, and insulin resistance that persists at 6 weeks postpartum [3]. After 10 to 12 years of the index-pregnancy, type 2 Diabetes Mellitus (DM2) was confirmed in 16.7% of MGH pregnant women [4].

Approximately one-third of the variation in fasting glucose in healthy non-diabetic and non-pregnant women is genetic [5]. Insulin directly regulates peripheral glucose uptake in muscle and adipose tissue [6]. The insulin pathway is mediated by a complex and highly integrated network that controls various processes. Considering the importance of insulin receptor substrate (IRS), especially IRS-1 on the insulin pathway, genetic alterations, such as polymorphisms, can alter the operation of this pathway. Common genetic variants at multiple loci are robustly associated with fasting glucose [7], DM2, and related glycemic traits [8,9]. Porzio et al. [10] described different frequencies of the insulin receptor substrate gene (IRS-1) polymorphisms between healthy (5%) and DM2 (10%–20%) subjects. IRS-1 is a highly polymorphic gene, located at 2q36, and its Gly972Arg variant is usually found in DM2 patients, especially in those who have insulin resistance, suggesting an important role of this polymorphism in the pathogenesis of diabetes [10]. The IRS-1 Gly972Arg variant is also associated with GDM. Tok et al. evaluated the polymorphism in 62 GDM women, and 14.5% presented this variant. Those patients with this gene polymorphism are obese in early pregnancy, and have high levels of glucose and fasting serum insulin [11]. Recently, we observed a higher frequency of the IRS-1 972Arg polymorphism in DM2 and GDM pregnant women, and also a direct link between this gene variant and obesity and insulin resistance [12]. Although several authors have demonstrated a relationship between the Gly972Arg polymorphism and diabetes, Vergotine et al. [13] showed that the Gly972Arg polymorphism was not associated with obesity, insulin resistance/sensitivity, or DM2 in the South African population.

The literature has also reported that diabetes hyperglycemia is related to increased DNA damage [14]. In fact, diabetes and hyperglycemia can generate reactive oxygen species (ROS) that may induce genetic damage. Some studies have shown increased DNA damage in leukocytes of type 1 and type 2 Diabetes Mellitus individuals [15], Pitozzi et al., [16] have previously described that patients with DM2 have a higher frequency of cells with DNA damage than those with DM1. Recently, Zengi et al., [17] also showed increased oxidative DNA damage in lean normoglycemic offspring of DM2 patients.

Diabetic patients have a higher frequency of the IRS-1 Gly972Arg variant and this polymorphism is directly related to insulin resistance and subsequent hyperglycemia. In diabetes, hyperglycemia and other associated factors generate reactive oxygen species that increase DNA damage. Since GDM and MHG pregnant women share some characteristics and induce similar effects in the offspring, we investigated if diabetic (DM2 even before pregnancy), gestational diabetic, and MGH pregnant women with the IRS-1 972Arg variant, might have higher levels of DNA damage. Therefore, the objectives of this study were to evaluate the presence of the IRS-1 Arg972 polymorphism in diabetic and mild gestational hyperglycemic pregnant women, and in their newborns. We evaluated the level of primary DNA damage in lymphocytes in these pregnant women. Additionally, we evaluated the relationship between the amount of genetic damage and the presence of the polymorphism.

The presence of the polymorphism in women with MGH and diabetes was relatively more common, although there was no statistically significant difference. Possibly due to insulin treatment, diet and exercises in MGH and diabetic groups and the non-diabetic group is at high risk for gestational diabetes mellitus, the DNA damage in MGH and diabetic groups were lower than the non-diabetic group.

## Methods

### Casuistry

The study protocol was approved by the Research Ethics Committee of Botucatu Medical School number (OF 545/2004) and all participants provided written informed consent.

The investigation was undertaken at the Diabetes and Pregnancy Tertiary Center, at the Botucatu Medical School, UNESP, Brazil. Eligible pregnant women underwent positive screening for GDM, including fasting glycemia level ≥90 mg/dL and risk factors for GDM in the late second trimester, according to the Brazilian Health Ministry recommendations, followed by referral for a diagnostic oral standard 75-g glucose tolerance test (OGTT) and a glycemic profile between the 24th and 28th weeks. GDM was confirmed when an abnormal screening test was followed by two or more values on a standard 75-g OGTT using the Carpenter and Coustan criteria [18]. MGH was diagnosed when an abnormal screening was followed by normal values of OGTT and the altered glycemic profile was characterized by fasting glycemia higher than 90 mg/dl and/or postprandial glycemia higher than 130 mg/dl [2,19]. Pregnant women with DM2 came to the service with a prior diagnosis. Inclusion criteria were as follows: classified in the study groups, gestational age of entry into the treatment protocol of 30 weeks for MGH and 20 weeks for type 2 DM, prenatal care and birth on the service, and consent form signature. DM1 patients were not included in this study. Exclusion criteria were multiple pregnancies, fetal malformations, congenital malformations, and gestational age at delivery lower than 34 weeks.

A total of 226 pregnant women were stratified into the following three groups: non-diabetic group, normal OGTT and glycemic profile (n = 62); mild gestational hyperglycemia, normal OGTT and abnormal glycemic profile (n = 63); and diabetic, abnormal OGTT and glycemic profile (n = 101). A total of 85 newborns were also evaluated: 31 newborns of mothers with normal glucose tolerance (non-diabetic group), and 12 from MGH and 42 from diabetic mothers.

For maternal hyperglycemia regulation, pregnant MGH and GDM women were treated with diet, physical exercise, and insulin therapy (if necessary) after the diagnosis, and women with type 2 diabetes were treated since before the beginning of pregnancy [20,21].

### Anthropometric and biochemical analyses

The body mass index (BMI) was calculated by body weight divided by the square of height. Plasma glucose was measured using the glucose oxidase method (Glucose Analyzer II Beckman®, Fullerton, California, USA). Glycated hemoglobin (HbA1c) was analyzed by HPLC (high-performance liquid chromatography; D10TM Hemoglobin Testing System, BIO RAD® laboratories, Hercules, CA, USA). The glycemic mean was calculated by the arithmetic mean of plasma glucose measured in all glycemic profiles performed at diagnosis. Large for gestational age (LGA) was defined as neonatal birth weight ≥ 90th percentile for gestational age (the weight/gestational age), and sex was determined according to service protocol.

### Gene polymorphism and DNA damage

From week 34 of gestation and before the onset of labor, maternal blood samples (5–10 mL) were collected into tubes with EDTA (Vacutainer®). From newborns, a blood sample (5 mL) was collected from the umbilical cord vein, also into tubes with EDTA (Vacutainer®). For gene polymorphism assays, a blood aliquot was stored at −20°C for subsequent DNA extraction, according to Salazar et al. [22]. The extracted DNA was maintained at −20°C until gene amplification by PCR. The concentration of DNA was measured in a spectrophotometer, and the integrity was determined by direct agarose gel electrophoresis. The Gly972Arg genotype was verified by the PCR-restriction fragment length polymorphism (RFLP) technique. A 263-base pair (bp) fragment containing the Gly972Arg segment was amplified with primers described by Almind et al. [23]: IRSF (5′ CTTCTGTCAGGTGTCCATCC 3′) and IRSR (5′ TGGCGAGGTGTCCACGTAGC 3′). The amplified fragment was confirmed by 6% polyacrylamide gel electrophoresis stained with silver nitrate. After confirmation of amplification, 263-bp PCR products were digested with 3 U of BstNI (New England Biolabs, Ipswich, MA, USA). The products of enzymatic digestion were analyzed by 6% polyacrylamide gel electrophoresis stained with silver nitrate and using a DNA molecular size marker. The fragments had the following sizes: 159, 81, and 23 bp in Gly972 homozygotes; 159, 108, 81, 51, and 23 bp in Gly/Arg972 heterozygotes; and 108, 81, 51, and 23 bp in Arg972 homozygotes.

The comet assay was used to evaluate primary DNA damage. Blood lymphocytes were isolated by Ficoll® gradient, and the assay was performed as in [24]. The lymphocytes (20 μl) were mixed with low melting point (LMP) agarose (120 μl), placed on pre-coated slides with normal melting point (NMP) agarose, and immediately covered with coverslip. The slides were left at 4°C for 10 min to solidify the agarose. The coverslip was gently removed and slides were immersed into an ice-cold freshly prepared lysis solution (2.5 M NaCl, 100 mM EDTA, 10 mM Tris, with 1% Triton 100-X, and 10% dimethyl sulfoxide). As a positive control, lymphocytes from healthy individuals were treated in vitro with H2O2 (200 μM), and incubated on ice for 30 min according to the protocol described by Blasiak et al. [25]. After lysis, the slides were placed onto a horizontal electrophoresis unit filled with fresh electrophoresis alkaline buffer (300 mM NaOH and 1 mM EDTA, pH >13). The alkali unwinding duration was 20 min. Electrophoresis was conducted at 4°C for 30 min at 25 V/cm and 300 mA. All the steps were carried out under minimal illumination. The slides were neutralized in a buffer (0.4 M Tris at pH 7.5) and fixed with absolute alcohol. The slides were stained with ethidium bromide (20 μg/ml in distilled H2O; 50 μl/slide), and analyzed in a fluorescence microscope connected to a charge-coupled device (CCD) camera and a personal computer-based analysis system (Comet Assay IV, Perceptive Instruments, UK) in order to determine the extent of DNA damage. Tail intensity (% of migrated DNA in the tail) was used do express DNA damage. One hundred randomly selected cells (50 from each of two replicate slides) were scored per blood sample.

### Statistical analysis

Analysis of variance (ANOVA), followed by Tukey’s multiple comparison test, was used for analyzing the characteristics of the study population (pregnant women and newborn). The Chi-square test was used to compare the frequencies of genotypes between the groups (pregnant women and newborns) and also for newborn weight classification. A two-factor interaction model was used in the associations. For DNA damage among the experimental groups, Gamma distribution was applied because the data presented non-normal distribution. Logistic regression was applied in the association between DNA damage and genotype. The level of statistical significance adopted was p < 0.05.

## Results

Table 1 shows the characteristics of the pregnant women (age, glycemic mean, initial and final BMI, and HbA1c) and newborns (plasma glucose and weight classification). For pregnant women, age, and initial and final BMI did not differ between the groups. MGH and diabetic groups showed higher glycemic means compared with the non-diabetic group (p > 0.05). Additionally, the glycemic mean was higher in the diabetic group than in the MGH group (p < 0.05). Diabetic pregnant women presented higher HbA1c when compared to those from the non-diabetic and MGH groups (p < 0.05). In newborns, plasma glucose concentrations were not different between the groups (p > 0.05). Regarding weight, the MGH group presented a lower frequency of newborns with adequate body weight for gestational age (AGA) than the non-diabetic and diabetic groups (p < 0.05). LGA newborns were more frequent in the MGH and diabetic groups than in the non-diabetic group (p < 0.05), and were more frequent in the MGH group than the diabetic group (p < 0.05).

**Table 1:** **Characteristics of study population**

	**Pregnant women**		
	**Non-diabetic**	**MGH**	**Diabetic**
**Number of subjects**	62	63	101
**Age** (years)	29.70 ± 5.50	31.50 ± 4.20	31.30 ± 5.40
**Glycemic Mean** (mg/dL)	80.56 ± 8.76	97.69 ± 7.85*	108.50 ± 15.82* ^**#**^
**Initial BMI** (Kg/m^2^)	29.34 ± 8.39	31.93 ± 9.43	32.49 ± 8.29
**Final BMI** (Kg/m^2^)	33.82 ± 8.01	36.17 ± 7.91	36.12 ± 6.62
**HbA1c**	5.45 ± 0.53	5.74 ± 0.67	6.33 ± 0.90* ^**#**^
		**Newborns**	
	**Non-diabetic**	**MGH**	**Diabetic**
**Number of subjects**	31	12	42
**Glycemia** (mg/dL)	64.76 ± 19.45	66.73 ± 22.59	69.56 ± 31.84
**Weight Classification** ^**a**^			
**SGA**	03 (10.0%)	00 (0.0%)	03 (7.3%)
**AGA**	27 (90.0%)	8 (66.6%)^αβ^	34 (82.9%)
**LGA**	0 (0.0%)	4 (33.3%)^αβ^	4 (9.7%)^α^

MGH: Mild gestational hyperglycemia.

BMI: Body Mass Index, HbA1C: Glycated Hemoglobin, SGA: small for gestational age.

AGA: adequate for gestational age, LGA: large for gestational age.

Data presented as mean ± standard deviation.

*p < 0.05 – statistically significant compared to non-diabetic group (Tukey’s multiple comparison test).

#p < 0.05 – statistically significant compared to MGH group (Tukey’s multiple comparison test).

^α^p < 0.05 – statistically significant compared to newborns of non-diabetic group (Chi-square test).

^β^p < 0.05 – statistically significant compared to newborns of diabetic group (Chi-square test).

The prevalence of the Gly/Arg heterozygote genotype was 12.90% (8/62), 19.5% (12/63), and 14.85% (15/101) in women from the non-diabetic, MGH, and diabetic groups, respectively. No statistically significant differences between the groups were detected (p = 0.61; Table 2). Only one pregnant woman from the non-diabetic group was homozygous for the Gly972Arg polymorphism. In newborns, the frequency of Gly/Arg was significantly (p < 0.05) higher in the MGH (16.67%) and diabetic groups (23.81%) than in the non-diabetic group (0.0%) (Table 2). Genotype distribution in both pregnant women and newborns was according to Hardy–Weinberg equilibrium.

**Table 2:** **Distribution (%) of the** ***IRS-1*** **Arg972 gene polymorphisms in pregnant women and newborns**

	**Pregnant women**		
	**Non-diabetic**	**MGH**	**Diabetic**
**Genotype**			
**Gly/Gly**	54 (87.10%)	51 (80.95%)	86 (85.15%)
**Gly/Arg**	08 (12.90%)	12 (19.05%)	15 (14.85%)
**Allele Frequency** ^a^			
**“G” Allele**	93.5%	90.4%	92.5%
**“A” Allele**	6.5%	9.6%	7.5%
**Total**	62	63	101
	**Newborns**		
	**Newborn of non-diabetic**	**Newborn of MGH**	**Newborn of diabetic**
**Genotype**			
**Gly/Gly**	31 (100.0%)	10 (83.33%)	32 (76.19%)
**Gly/Arg**	0 (0.0%)	2 (16.67%)*	10 (23.81%)*
**Allele Frequency** ^a^			
**“G” Allele**	100.0%	91.6%	88.1%
**“A” Allele**	0.0%	8.4%	11.9%
**Total**	31	12	42

MGH: Mild gestational hyperglycemia.

Data presented as N (%).

^a^Data presented as %.

*p < 0.05 compared to non-diabetic group (Chi-square test).

Table 3 shows the amount of DNA damage (tail intensity) in pregnant women. A lower level of damage was observed in the diabetic group compared to the non-diabetic group (p < 0.05). Table 4 shows the association between tail intensity and the Arg972 genotype in pregnant women, highlighting the genotype and the groups. The logistic regression showed no significant association (p > 0.05).

**Table 3:** **DNA damage (tail intensity) in pregnant women**

	**Groups**		
	**Non-diabetic n = 39**	**MGH n = 24**	**Diabetic n = 47**
**Tail Intensity Mean %** ± **SD**	38.81 ± 38.13	37.59 ± 36.28	36.19 ± 37.29*

MGH: Mild gestational hyperglycemia.

*p = 0.0064 compared to non-diabetic group (Gamma distribution).

**Table 4:** **DNA damage (tail intensity) according to the** ***IRS-1*** **Arg972 considering pregnant women study groups**

**Groups**	**Genotype**	**Tail intensity mean ± SD**
**Non-diabetic**	GG (n = 35)	38.6 ± 17.0
	GA (n = 04)	36.06 ± 20.2
**MGH**	GG (n = 21)	38.32 ± 13.5
	GA (n = 03)	30.16 ± 11.6
**Diabetic**	GG (n = 40)	34.7 ± 11.3
	GA (n = 07)	43.3 ± 13.3

MGH: Mild gestational hyperglycemia.

No statistical difference (logistic regression).

## Discussion

The present study aimed to evaluate the presence of the Arg972 polymorphism of the IRS-1 gene in diabetic pregnant women or those with mild gestational hyperglycemia, as well as in their newborns, and to evaluate and correlate with genotoxicity in these pregnant women. Genotype frequencies did not differ significantly between the groups of pregnant women, but the presence of the polymorphism was relatively more common in women presenting mild gestational hyperglycemia and diabetes (gestational and overt diabetes).

Orkunoglu *et al.* [26] found a similar frequency of polymorphisms in 186 Turkish individuals with BMI <25, *i.e*., 12.90% in control subjects and 14.2% in type 2 diabetics. Moreover, in a meta-analysis, it was concluded that 8.6% of healthy subjects and 11.4% of patients with non-insulin dependent diabetes *mellitus* (NIDDM) had this polymorphism with an odds ratio of 1.25 (95% CI 1.05–1.48) [27]. Tok *et al*. [11] found Arg972 in14.5% of patients with GDM and confirmed that this variant may define a subgroup of patients who tended to be obese. This suggests that it might be related to insulin resistance, which is seen in obese patients with GDM. Falluca *et al*. [28] verified a frequency of 11.0% of the polymorphism in women with GDM, 9.8% in gestational impaired glucose tolerance (G-IGT), and 7.9% in women with a negative glucose-challenge test (NGT) result, suggesting that the IRS-1 genetic polymorphism is involved in the occurrence of gestational diabetes, as well as type 2 diabetes mellitus. After comparison of our findings with the literature data, it was observed that the frequency of this polymorphism is higher in our study population, although this was not statistically different.

The mild gestational hyperglycemia pregnant women presented the highest frequency of the polymorphism, although without statistical difference. Negrato *et al*. [29] demonstrated that patients with MGH showed higher HOMA-IR (index for insulin resistance evaluation) values and lower peripheral insulin sensitivity (although this was not a statistically significant difference) compared with the normal glucose tolerance group, suggesting that besides presenting insulin resistance, they may also have a compensatory hyperinsulinemia. There is evidence of the relationship between the presence of the polymorphism Arg972 and insulin resistance, where the individuals with the polymorphism present low levels of fasting glucose and C-peptide, as well as impaired insulin synthesis and secretion in the presence of glucose [23,30,31]. Shirakami *et al*. [32] showed that in obese mice, IRS-1 knockout produces severe insulin resistance and lower expression of insulin receptor substrate (IRS-1 and IRS-2).

Regarding DNA damage, the comet assay was used to evaluate basal DNA damage levels in pregnant women with either diabetes or mild hyperglycemia. The DNA damage levels were lower in the diabetic group compared to the non-diabetic group. In the non-diabetic group, who showed risk factors for gestational diabetes mellitus, OGTT and GP indicated no hyperglycemia or change in glucose metabolism. Major risk factors presented in this group included overweight and obesity, which may be associated with a sedentary lifestyle and an unbalanced diet. Availability of transport, devices designed to facilitate daily activities, easy food access, and the fact that high-fat and high-sugar foods are frequently the cheapest, have created an “obesogenic” environment [33], which may be one factor to explain the increased levels of basal DNA damage observed here. Karbownik-Lewinska *et al*. [34] showed that overweight and obesity in adults are directly associated with increased oxidative damage to macromolecules.

Pregnant women in the MGH and diabetic groups also show the same risk factors as the non-diabetic group, however, MGH and diabetic groups underwent medical monitoring and treatment during pregnancy. The treatments applied to these pregnant women included physical activity, diet, and insulin therapy as needed. Pregnant women of the MGH and diabetic groups were diagnosed between the 24th and 28th weeks of gestation, and blood samples were collected from the 36th week, and therefore, we have a minimum of 8 weeks of such treatments. Due to the reduced need, only a small percentage of pregnant women with MGH were treated with insulin during pregnancy, and this may be the reason for basal DNA damage are not as lower as the pregnant women of diabetic group, which besides diet and exercises, most were treated with insulin. Supporting this hypothesis, Wayhs *et al*. [35] showed significant reductions in the DNA damage index in diabetic animals treated with insulin, suggesting that treatment with insulin protects against oxidative DNA damage.

Wang and Huang (2005) [36] observed that moderate exercise attenuates lymphocyte apoptosis induced by oxidative stress, possibly by improving intracellular antioxidative capacity. Kim *et al*. [37] showed that 9 weeks of training with three types of exercise increased the activity of antioxidant enzymes and consequently decreased the lipoperoxidation, which was verified by reduced plasma MDA concentration and lymphocyte DNA damage. Siu *et al*. [38] also confirmed that 8 and 20 weeks of habitual exercise conferred increased resistance of rat lymphocytes to oxidant-induced DNA damage. Diet and lifestyle can also contribute to the reduction of DNA damage. Staruchová *et al*. [39] suggested that a well-balanced food consumption with higher fruit and vegetables intake has a protective effect against oxidative damage. Ochi & Sakai [40] showing that oxidative stress can be greatly reduced by the improvement of lifestyle, such as diet. These facts show that despite these groups of pregnant women presenting diabetes and mild gestational hyperglycemia, the medical monitoring and treatment they received during pregnancy contributed to the decreased levels of basal DNA damage.

In the present study, there was no association between the presence of the Arg972 polymorphism of the *IRS-1* gene and the basal DNA damage in these groups of pregnant women. In addition to the presence of the polymorphism not differing in the pregnant group, the basal DNA damage level in the diabetic group was reduced compared with the non-diabetic group possibly due to medical monitoring and treatment this group received during pregnancy.

Although our newborn sample size is small, children of the MGH and diabetic groups showed a higher prevalence of the Arg972 polymorphism compared with children of the non-diabetic group. Despite the polymorphism not differing among the mothers, their newborns of MGH and diabetic mothers presented a higher prevalence of polymorphism. We did not find manuscripts discussing the polymorphism frequency in newborns of diabetic mothers to compare and discuss this data, but, Bezerra *et al.*, [41,42] shows a frequency of 12.9% of the Arg972 polymorphism in healthy newborns, confirming the higher polymorphism frequency in newborns of MGH and diabetic mothers found in our manuscript. With the result of the polymorphism evaluation in newborns, it is necessary to monitor the development of these childrens more closely and intervention preventively to control the negative effects that the presence of this polymorphism can bring. Some studies have shown that the presence of the Arg972 polymorphism in newborns is associated with decreased birth weight, lower body length and head circumference in neonates, reinforcing the hypothesis that genetically determined insulin resistance and/or reduced insulin secretion can result in impaired insulin-mediated growth in the fetus [41,42]. There is a close relationship between this polymorphism and DM2 [31], DMG [11,28], obesity [43-45], and insulin resistance [12,23,31,32]. In addition, newborns of the MGH and diabetic groups had a higher frequency of large body weight for gestational age. Rudge [46] showed that newborns of women with MGH presented 53.8% for macrosomia, a proportion similar to the 51.9% observed in gestational and overt diabetes. The perinatal mortality rate is 41‰, which is similar to diabetic pregnant women and ten-fold higher than normal glucose tolerance pregnant women [21]. The attributable risk of perinatal death in this group is 4.16%, which is comparable to that identified in groups of diabetic pregnant women [47].

There was no statistical difference between the genotypes of pregnant women. When the relationship between the polymorphism and basal DNA damage was evaluated, it was not possible to establish an association. The polymorphism was more prevalent in newborns of diabetic and MGH women. We believe that it is necessary to increase the number of subjects to be examined in order to better determine the biological role of the Arg972 polymorphism in these patients. Despite being classified as low-risk, pregnant women with mild gestational hyperglycemia characterize a population of maternal and perinatal adverse outcomes, and that, together with their newborns, require better monitoring by professionals and health services.
